# Supplementary material for: High-Throughput Proteomic Profiling of Nipple Aspirate Fluid from Breast Cancer Patients Compared with Non-Cancer Controls: A Step Closer to Clinical Feasibility
Source: J Clin Med. 2021 May 21;10(11):2243. doi: 10.3390/jcm10112243 (PMC8196703; doi:10.3390/jcm10112243)
Supplement: Supplementary file 1 [file jcm-10-02243-s001.zip › Supplementary data/Supplementary Figures.docx]

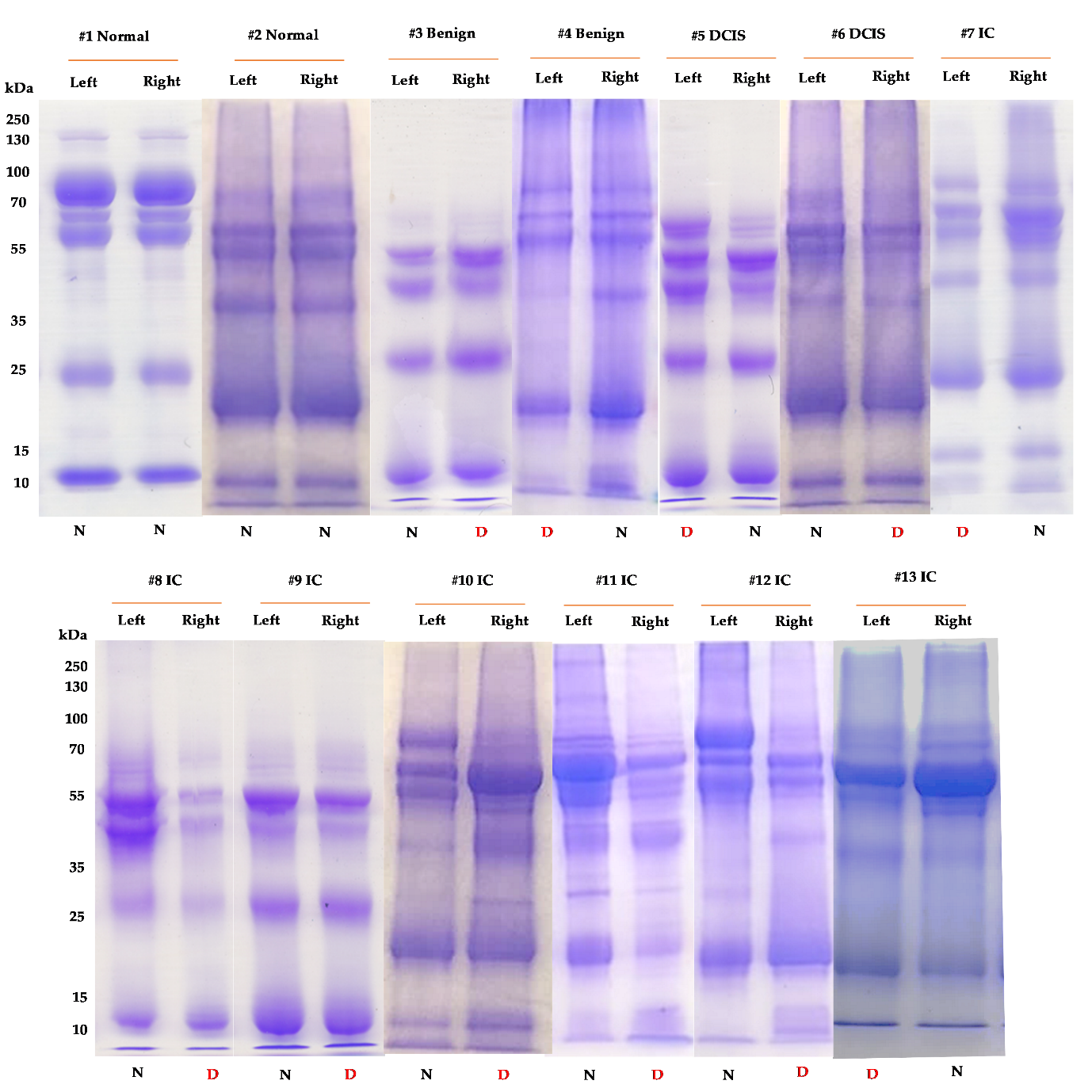
Supplementary Figure S1

**Figure S1 -** SDS-PAGE of paired NAF samples, DCIS – ductal carcinoma in situ, IC – invasive carcinoma, N – normal, D - disease.

Supplementary Figure S2


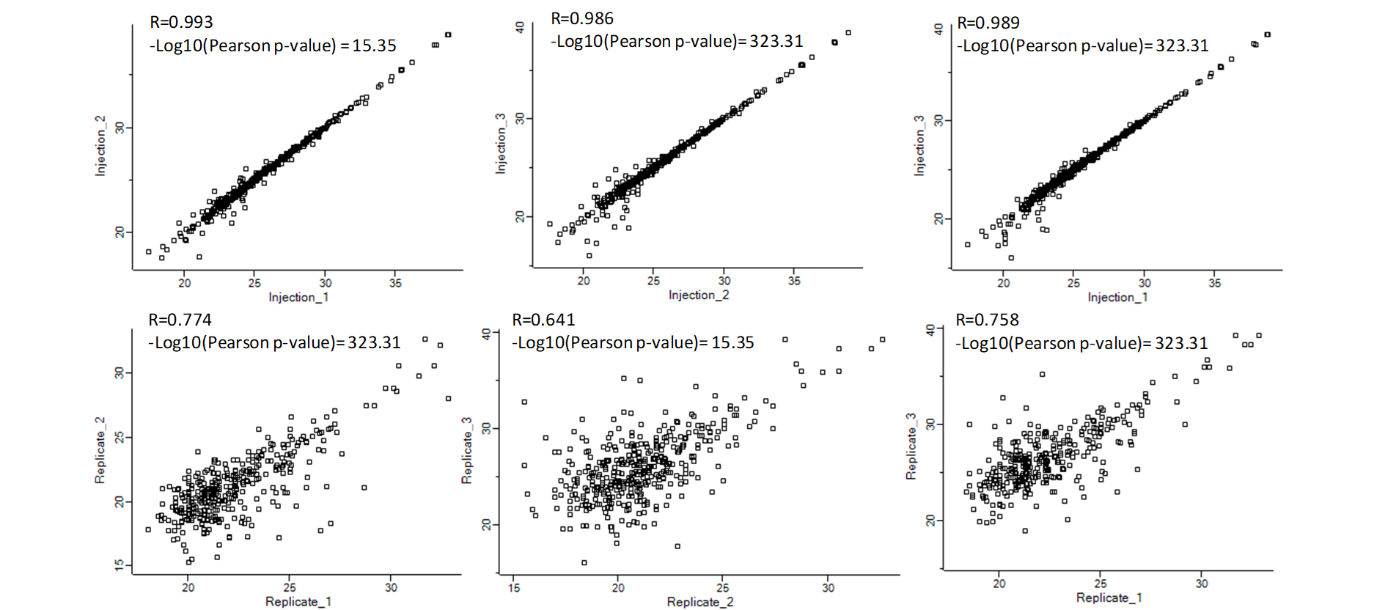


**Figure S2 -** Scatter plots comparing Log2 annotated protein groups from biological (Replicate_1,2,3) and technical replicates (Injection_1,2,3) of subject 9 left breast sample indicate that both the sample preparation and instrument method provide high concordance between identically processed samples; Pearson correlation coefficient (R) and –Log10(Pearson correlation p-values) labelled
